# Leukotoxicity after moderately Hypofractionated radiotherapy versus conventionally fractionated dose escalated radiotherapy for localized prostate Cancer: a secondary analysis from a randomized study

**DOI:** 10.1186/s13014-019-1223-2

**Published:** 2019-01-30

**Authors:** Giuseppe Sanguineti, Diana Giannarelli, Maria Grazia Petrongari, Stefano Arcangeli, Angelo Sangiovanni, Biancamaria Saracino, Alessia Farneti, Adriana Faiella, Mario Conte, Giorgio Arcangeli

**Affiliations:** 1 Department of Radiation Oncology, IRCCS Regina Elena National Cancer Institute, Via Elio Chianesi 53, 00144 Rome, Italy; 2 Biostatistical Unit, IRCCS Regina Elena National Cancer Institute, Rome, Italy

**Keywords:** Leukotoxicity, Conventional radiotherapy, Hypofractionated radiotherapy, Localized prostate cancer

## Abstract

**Background:**

To compare WBC counts during treatment of localized prostate cancer with either conventionally fractionated (CF) or moderately hypofractionated (HYPO) radiotherapy.

**Methods:**

Weekly blood test results were extracted from the charts of patients treated within a phase III study comparing HYPO to CF. In order to compare WBC counts at the same nominal dose in both arms and thus to tease out the effect of fractionation, for each recorded WBC value the corresponding cumulative total dose was extracted as well. WBC counts were binned according to percentiles of the delivered dose and three dose levels were identified at median doses of 16, 34.1 and 52 Gy, respectively. A General Linear Model based on mixed design Analysis Of Variance (ANOVA) was used to test variation of WBC counts between the two treatment arms.

**Results:**

Out of 168 randomized patients, 140 (83.3%) had at least one observation for each one of the selected dose levels and were included in the analysis. Mean counts were lower in the CF than the HYPO arm at all selected dose levels, reaching a statistically significant difference at dose level #3 (5397/mm^3^ vs 6038/mm^3^ for CF and HYPO, respectively, *p* = 0.004). The GLM model confirms that the impact of dose on WBC counts is significantly lower in the HYPO arm over the CF one (Greenhouse-Geisser test, *p* = 0.04). Interestingly, while WBC counts tend to drop throughout all dose levels in the CF arm, this is the case only in the earlier part of treatment in the HYPO arm.

**Conclusion:**

This secondary analysis of a phase III study shows that dose fractionation is correlated to WBC drop during treatment of localized prostate cancer, favoring HYPO over CF.

**Electronic supplementary material:**

The online version of this article (10.1186/s13014-019-1223-2) contains supplementary material, which is available to authorized users.

## Background

Ionizing radiations cause profound depression of the hematopoietic system [1]. Several clinical studies have shown a drastic decreases in white blood cell (WBC), red blood cell and platelet counts during radiotherapy (RT) [2–9].

Historically the issue of bone marrow depression during a course of external beam RT has been managed by weekly checks of blood counts, though several studies have shown that the risk of severe (grade 3) or even mild (grade2) hematological (HEM) toxicity is remarkably low, questioning the widespread need for such costly tests [2, 8, 9]. More recently, the attention has been shifted towards the detrimental prognosis of leukopenia (and in particular lymphopenia) in patients affected by various solid cancers both before and during RT [10–14]. Acute leukopenia often protracts well beyond treatment [7] justifying the attempt to avoid HEM depression at the time of RT [15].

The effects of radiation on circulating blood cells depend on the total dose of RT administered, the irradiated volume, the primary tumor location, pretreatment blood indices, as well as other concurrent therapies received by the patient [2, 6–9]. Few studies have specifically investigated predictive factors of HEM depression in patients with localized prostate cancer undergoing external beam RT [2, 3, 6, 7]. Despite this burden of data, the effect of fraction size on blood cell counts during RT for prostate cancer has never been investigated in details.

The issue is relevant for two reasons. Firstly, there is an increasing awareness of the relationship between ionizing radiations and the immune response even in prostate cancer [16]; secondly, moderately hypofractionated RT (HYPO) at doses per fraction ranging from 2.8 to 3.2 Gy for localized prostate cancer is currently widely used as supported by multiple phase III studies [17–20].

The present study aims at clarifying the impact of HYPO compared to conventionally fractionated (CF) RT on WBC counts during treatment throughout a secondary analysis of a phase III study.

## Methods and materials

### Patients and treatments

All patients treated within a randomized, single-Institution trial were analyzed. The details of the trial have been previously published [18]. Briefly, patients with intermediate to high risk localized prostate cancer were treated with 9-month total androgen blockade (bicalutamide, 50 mg, daily and a luteinizing hormone-releasing hormone analog depot, monthly). At the 67th day from the first bicalutamide intake, patients started definitive radiotherapy, either 80 Gy in 40 fractions over 8 weeks, conventional fractionation (CF) arm, or 62 Gy in 20 fractions over 5 weeks, hypofractionated (HYPO) arm. Patients in the CF arm were treated five times per week while those in the HYPO arm, four times per week. In both arms, RT targeted the prostate and the whole seminal vesicles. A three-dimensional conformal radiotherapy (3D-CRT) technique with 6 coplanar 15 MV photon fields was used. No attempt was done to cover pelvic lymph nodes.

### Statistics

The protocol mandated weekly hematologic cell counts during treatment and here we focus on WBC counts only. Results of blood tests were usually transcribed in the RT chart. Therefore, data were obtained through chart review from the date of diagnosis up to the end of radiotherapy. Unfortunately, leukocyte differential counts were not systematically recorded.

Based on the date of blood sampling and each individual RT schedule, a number of fractions was associated to each WBC count. Then, the cumulative (nominal) radiation dose was obtained by multiplying the dose per fraction (either 2 Gy or 3.1 Gy as appropriate) by the number of fractions delivered. WBC counts were binned based on the cumulative total dose delivered up to the day of blood sampling, with baseline observations assigned a dose value of zero.

For statistical analysis, cell counts were treated as absolute ones. In case of multiple individual measurements within a given dose range, or when measurements from various time points were pooled, the average value was considered. In case of missing data, no attempt was done to interpolate between the available values. Therefore, missing data points were excluded.

Distributions of samples between groups were assessed with Pearson’s chi-square; means were compared with Student’s t test. A General Linear Model based on mixed design Analysis Of Variance (ANOVA) was used to test variation of WBC counts at increasing cumulative doses and between the two treatment arms. This model tests three hypotheses: 1. The one that no difference exists in mean WBC counts and dose (‘within effect’); 2. The one that no difference exists between the two groups (‘between effects’); 3. The one that possible differences according to dose are constant between the two groups (‘interaction effect’). Since in the GLM model patients with missing values are excluded, in order to minimize incomplete data loss, we preliminarily identified the cumulative dose intervals in which the majority patients had all data points. Moreover, since pre-treatment WBC counts were available only for a minority of patients, baseline data were not included in the GLM analysis. Statistical significance was claimed for *p* values less than 0.05.

## Results

### Patients and observations

As previously reported [18], 168 patients (85 and 83 in the CF and HYPO arms, respectively) were accrued from January 2003 to December 2007. Selected patient, tumor and treatment characteristics have been reported in details elsewhere [18]. All patients received the assigned prescribed nominal dose over an average (SD) time of 59.8 (5.7) and 33 (4.4) days in the CF and HYPO arms, respectively (*p* < 0.001).

WBC counts collected during treatment up to the nominal total dose of 62 Gy (*N* = 705) were divided into in 3 or 4 groups according to tertiles or quartiles of the delivered nominal total dose, respectively. We identified 140 patients (83.3%) and 109 patients (64.9%) with observations for all the 3 or 4 intervals, respectively. Given the large number of patients with missing data in the 4-tier approach, we decided to further analyze data based on the former sub-classification only. Of note, the median dose of intervals 1–3 was spaced by a very similar nominal dose, ≈18 Gy.

**Fig. 1:**
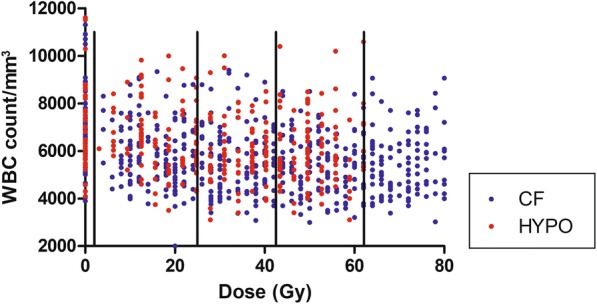
WBC counts (*N* = 835) for the selected 140 patients with at least one observation during each interval/dose level from 1 to 3 and by treatment arm (CF: conventional fractionation; HYPO: hypofractionation). Data are summarized in Table 1

Table 1 and Fig. 1 summarize observations for the 140 selected patients. Seventy-seven patients (55%) had baseline observations, with a non-dissimilar distribution between arms (*p* = 0.508). As expected, based on the larger number of fractions needed to reach a given dose for patients treated with conventional fractionation over hypofractionated RT, patients in the CF arm had a statistically higher number of observations within all treatment intervals, though the number of analyzed patients by arm is very close (*N* = 71 and 69 in the CF and HYPO arms, respectively) (Table 1).

**Table 1:**
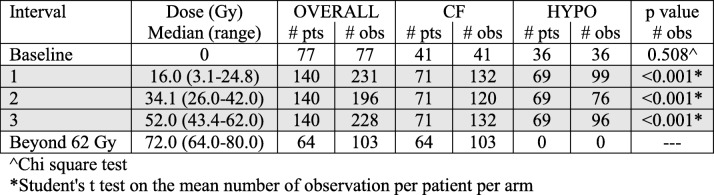
Number of patients and observations by interval/dose level. All selected patients (*N* = 140) have at least one observation during each interval from 1 to 3. The grey area corresponds to the General Linear Model (GLM) area of Fig. 2

^Chi square test

*Student’s t test on the mean number of observation per patient per arm

*Abbreviations: pts*. patients, *obs* observations, *CF* conventional fractionation, *HYPO* hypofractionationation

### WBC counts

As shown in Table 2, baseline WBC counts/mm^3^ averaged out at ≈7000. The largest drop in WBC counts was observed from baseline to the first dose interval (≈800 counts/mm^3^, − 11.1%, *p* < 0.001). The magnitude of the difference in average WBC counts from the 1st to the 2nd and from the 2nd to the 3rd interval was progressively smaller and lost statistical significance: − 6.3%, *p* = 0.018 and − 1.3%, *p* = 0.619, for the former and the latter time periods, respectively.

**Table 2:**
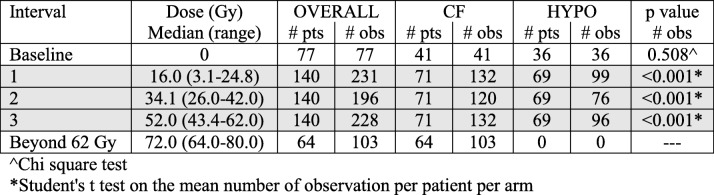
Mean (SD) White Blood Cell counts by interval/dose level. The grey area corresponds to the General Linear Model (GLM) area of Fig. 2

*Student’s t test

Abbreviations: see Table 1

### WBC counts by arms – GLM model

**Fig. 2:**
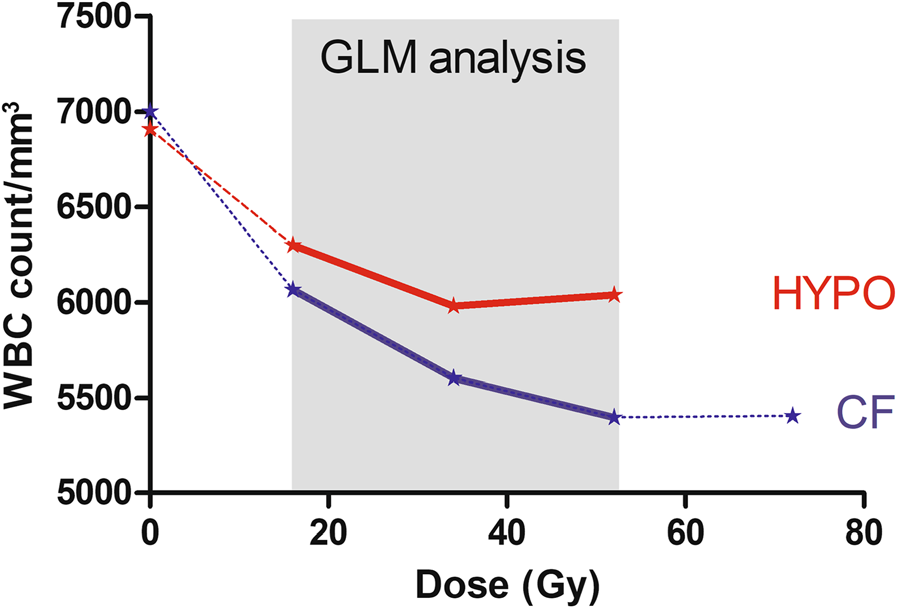
Average WBC count by interval/dose level and arm (CF: conventional fractionation; HYPO: hypofractionation). The grey area reports the results of the GLM analysis on 140 patients. Data are summarized in Table 2

Average absolute WBC counts per patient at selected time points are summarized in Table 2 and illustrated in Fig. 2. Baseline mean counts were not statistically different before treatment (*p* = 0.821), but diverged progressively reaching statistically significant *p* values at an average median delivered dose of 34.1 Gy (Table 2).

The GLM (mixed design ANOVA model) analysis was restricted to intervals 1–3, where all the selected patients had at least one observation for each dose level (Fig. 2, grey area). Since sphericity assumption was not met (Mauchly test, *p* = 0.001), the corrected test of Greenhouse-Geisser was considered. The model shows a main effect of dose on counts at different intervals (Greenhouse-Geisser test, *p* < 0.0001), reflected by a progressive decrease of average counts at increasing doses (Table 2). However, the impact of dose on WBC counts is different between arms (Greenhouse-Geisser test, *p* = 0.04), with less effect in the HYPO arm over the CF one. Also interaction between WBC counts and treatment arm resulted to be significant (*p* = 0.04) confirming the different behavior of WBC counts in each fractionation schedule.

Next we further analyzed the effect of dose on each separate arm. In the CF arm (Table 2 and Fig. 2), post-hoc analysis showed a highly significant reduction in average counts between interval 1 and both intervals 2 and 3 (both *p* < 0.0001 after Bonferroni correction). Moreover, there was a borderline difference in mean counts between intervals 2 and 3 after Bonferroni correction (*p* = 0.058). In the HYPO arm, we also observed a main effect of dose on counts (*p* = 0.020), though the only statistically different interval time was the one between 1 and 2 (*p* = 0.009 after Bonferroni correction).

## Discussion

The present study shows that moderate HYPO is associated with less acute leukopenia than CF. Within a given (cumulative) dose level, we found that fractionation increases the extent of WBC count drop. Although the trial was not designed to assess differences in WBC counts, this post-hoc analysis reveals a protective effect of moderate hypofractionation for localized prostate cancer on radiation-induced leukopenia, compared to CF. Since there is a loss of lymphoid tissue during ageing [21] along with a decline in immune functions [22] which might be implicated in the increased susceptibility of the elderly to a number of diseases, including cancer, our findings are noteworthy, considering that the median age of enrolled patients was 75 years (range 72–77).

Several studies have shown that external beam RT for tumors of various primary sites is associated with a 24–37% decrease in WBC counts by the end of treatment [4, 5, 7, 8, 23–25]. Baseline WBC counts have been found to be highly predictive of leukopenia during RT for various solid malignancies [3, 8, 9]. Moreover, leukopenia depends on both the dose delivered and the amount of bone marrow irradiated, with 40% + of bone marrow receiving at least 15 Gy considered a situation at risk of developing HEM toxicity [26]. Therefore, since baseline WBC levels [9] as well as irradiated volumes differ significantly among primary sites (with the pelvis and the vertebrae containing as much as 60% of the total bone marrow [27]), contemporary studies investigating the effect of RT on WBCs have focused on selected primary sites only [3, 6, 7, 13, 15, 28, 29]. Moreover, androgen deprivation therapy which is often used in addition to RT for localized prostate cancer has not been found to have a detectable effect of WBC counts during RT [2, 6], while chemotherapy is rarely indicated in this setting, both factors minimizing the confounding biases of ionizing radiation on bone marrow function.

While the risk of severe or higher (grade 3+, < 2000/mm^3^) WBC toxicity during a course of fractionated RT for localized prostate cancer is remote [3, 9], less intense effects are common and although within the normal range [16] they can persist for a long time after RT [3, 6]. It has been hypothesized that RT may have an immunosuppressive effect [16] and this in turn may increase the risk of tumor progression as shown in a variety of other solid tumors [10–14]. Therefore, though the clinical implications of mild to moderate leukopenia in prostate cancer are unknown, it seems desirable to adopt fractionation schemes associated with a lesser degree of leukotoxicity.

Previous studies have found irradiated volume [6] and baseline counts [3] to be independent predictors of leukotoxicity during RT for prostate cancer. In a prospective study on 113 patients undergoing IMRT for prostate cancer, Pinkawa et al. showed that the cumulative incidence of grade 2 leukopenia was 15% versus 2% with and without whole-pelvic (WP) RT, respectively [6]. Cozzarini et al. found baseline values to be the only independent predictor of WBC drop at the end of post-prostatectomy WP-IMRT of 125 prostate cancer patients. Of note, patients were treated with a relatively wide number of fractions (ranging from 28 to 43), but no (protective) effect was found for hypofractionation on leukotoxicity [3].

One major limitation of the present study is the lack of differential counts of WBC though this is common to other studies [8] and has limited impact in a comparative study like this one. The majority (> 90%) of circulating WBCs are either neutrophils (≈60–70%) or lymphocytes (≈30%). We did not systematically record the differential count of leukocytes and thus we cannot state which one, between neutrophils and lymphocytes is responsible for the observed (detrimental) effect of fractionation. The former ones arise from stem cells in the bone marrow, migrate in the blood stream and are short lived (life span of ≈ 5 days). Even if they are nucleated and thus potentially sensitive to the direct effects of ionizing radiations, doses up to 50 Gy are considered to have little if no effect on their count [30]. Therefore, radiation induced neutropenia is mostly due to the killing of (bone marrow) progenitor cells which are known to be more sensitive to RT [31]. Interestingly, according to experiments from a variety of animal studies using a range of multifraction regimens, normal bone marrow cells behave like acutely responding tissues with little effect of fraction size consistently with a poor repair capacity of sublethal damage between fractions [32]. In one of these studies, Tarbell et al. actually found a significantly steeper bone marrow survival curve after multiple daily fractions of 1.2 Gy rather than single or twice daily doses of 2.0 Gy [33], suggesting that fractionation may have a detrimental rather than a protective effect on cell survival.

Also lymphocytes arise from progenitor cells in the bone marrow, but maturate elsewhere remaining one of the most radiosensitive mammalian cells [34]. Interestingly, only 10–15% of lymphocytes are distributed in the bone marrow [35, 36] and, in order to perform antigen surveillance of the whole body, lymphocytes keep trafficking among different anatomical sites [37]. These distinct features explain why lymphopenia has been found after treatment of tissues that do not contain either bone marrow or lymphatics, as the brain [28, 38]. In one of these experiments, MacLennan et al. showed that the log-level of lymphopenia shortly after a given total dose of cranial irradiation was linearly dependent upon the number of fractions into which the irradiation was divided [38]. In one recent study on post-mastectomy radiotherapy, the Authors were able to detect a significantly higher degree of lymphopenia after 50 Gy at 2 Gy fraction rather than 40.3 Gy in 13 fractions. However, the difference between the two arms was detected 6 months after the end of treatment and not during or immediately after treatment completion [39].

Therefore, both clinical and experimental data are suggestive of a detrimental effect of fractionation on both neutrophil and lymphocyte counts, possibly due to reassortment of cells in the cell cycle and redistribution of circulating cells, respectively [33, 38]. Interestingly, in our experience, the fractionation effect overcame the slightly higher weekly dose rate in the HYPO arm over the CF one (3.2 Gy × 4, 12.8 Gy/week vs 2 Gy × 5, 10 Gy/week in the HYPO and CF arms, respectively), that would be supposed to negatively affect the survival of acutely responding cells [40].

In the present study, the difference in leukotoxicity between fractionation schemes was detected in the setting of limited pelvic volumes irradiation (prostate and seminal vesicles) and of conformal radiotherapy. Even if this do not represent a methodological limitation of the study, may narrow the applicability of the present results. Moreover, at present there is no conclusive evidence that the dose to (sub-regions of) the bone marrow is responsible for acute WBC toxicity for prostate cancer patients, but there is only a generic evidence of an increased WBC toxicity at enlarging irradiated volumes [6, 7]. Therefore, the effect of treatment technique (3DCRT vs IMRT/VMAT) on HEM toxicity for prostate cancer remains undetermined and likely related to the amount of either bone marrow or circulating white blood cells that are incidentally irradiated.

Future work will need to elucidate the WBC subtype responsible for the ‘fractionation effect’ observed in the present paper and its duration. Emerging data show that the various lymphocyte subpopulations have different sensitivity to ionizing radiations [39, 41–43] with potential implications on both outcome [43] and treatment strategy [16] for localized prostate cancer.

## Conclusion

The present data show that moderate hypofractionation for localized prostate cancer is associated with less WBC count depression during treatment than conventional fractionation. Along with the reduction in the number of treatment sessions, these findings add to the attractiveness of HYPO and enhance its favorable cost-effectiveness profile, especially in light of the increasing number of elderly patients in need of care.

## Additional file

Additional file 1: Original research protocol. (DOC 250 kb)

## Abbreviations

3D-CRT: Three-dimensional conformal RT
CF: Conventionally fractionated RT
GLM: General Linear Model
HEM: Hematological
HYPO: Moderately hypofractionated RT
IMRT: Intensity modulated RT
RT: Radiotherapy
VMAT: Volumetric modulated arc therapy
WBC: White blood cells
WP: Whole pelvic
