# Supplementary material for: Leukotoxicity after moderately Hypofractionated radiotherapy versus conventionally fractionated dose escalated radiotherapy for localized prostate Cancer: a secondary analysis from a randomized study
Source: Radiat Oncol. 2019 Jan 30;14:23. doi: 10.1186/s13014-019-1223-2 (PMC6352380; doi:10.1186/s13014-019-1223-2)
Supplement: Supplementary file 1 — Original research protocol. (DOC 250 kb) [file 13014_2019_1223_MOESM1_ESM.doc]

**Mono-institutional Prospective Phase III Randomized Study of Hypofractionated Radiotherapy in combination with Total Androgen Deprivation in High Risk Prostate Cancer**

First version, November 2002.

Writing Committee: Giorgio Arcangeli, Stefano Arcangeli, Lidia Strigari

Principal investigator:

Giorgio Arcangeli

Radiotherapy Dept.

Regina Elena Cancer Inst

Via E. Chianesi 53

00144-Rome, Italy

Tel ++39 06 52666131

Fax ++39 06 52665667

e-mail: [arcangeli.gio@tiscali.it](mailto:arcangeli.gio@tiscali.it)

Participants:

Giorgio Arcangeli*, Stefano Arcangeli, Sara Gomellini, M.Grazia Petrongari, Bianca Saracino, Lidia Strigari

CONTENTS

01 Background and rationale……………………………3

02 General remarks……………………………………...3

2.1 Definition of high risk tumors……………………………………...3

2.2 Treatment…………………………………………………………..3

03 Trial objective…………………………………...…...4

3.1 Primary……………………………………………………………..4

3.2 Secondary…………………………………………………………..4

04 Patient selection criteria……………………………...4

4.1 Inclusion criteria……………………………………………………4

4.2 Exclusion criteria…………………………………………………...4

05 Study design………………………………………….4

06 Therapeutic regimens………………………………...5

6.1 Hormonal therapy…………………………………………………..5

6.2 Radiation therapy…………………………………………………...5

07 Randomization………………………………………..6

7.1 Arm A (control)……………………………………………………..7

7.2 Arm B (experimental)……………………………………………....7

7.3 Randomization procedures……………………………………….....8

08 Evaluation…………………………………………….8

8.1 Endpoints…………………………………………………………....8

8.2 Timing………………………………………………………………9

09 Disease monitoring and treatment

of progressive disease…………………………………………...10

9.1 Disease monitoring…………………………………………………10

9.2 Treatment of progressive disease…………………………………..11

10 Statistical consideration, data analysis, reporting……11

11 Ethical-legal considerations………………………….12

12 Administrative issues………………………………...12

12.1 Planning documentation…………………………………………..12

13 References…………………………………………….12

APPENDIX

BLANC TOXICITY FORMS

PATIENT FORMS

**01 Background and rationale**

Combined analysis of patient outcome after both external beam radiotherapy (RT) and brachytherapy has recently led to the assumption that the α/β ratio of prostate cancer is lower than for most other tumors and approaches a value characteristic to normal late responding tissues. Values between 1.2 and 4 Gy have been proposed (1-8). Therefore, to deliver an equivalent biological dose (BED) to the prostate using fewer and larger than conventional 2 Gy fractions (hypofractionation), the total dose must be proportionally reduced. If the α/β ratio is lower in the tumor than in the surrounding late responding normal tissues, the equivalent total dose delivered to the former will translate in a significant reduction of the total equivalent 2 Gy dose absorbed by the latter, with a potential decrease in the late side-effects. Hypofractionation would also reduce the acute side-effects providing the overall time would be sufficiently long to allow the repopulation of the early responding normal tissues.

Fraction sizes > 2 Gy have already been used by other groups which reported results that seemed to be comparable to conventional schedules (9-12). These observation were partially confirmed by 2 recent randomized trials on T1-T2 prostate cancer (13,14), which reported similar late toxicity rates between the short and conventional schedule. However, the 5-year biochemical failure rate in the Canadian study (13) was significantly higher in the hypofractionation in comparison to standard fractionation arm (59.95% vs 52.95%), likely because of the non biological equivalence of the 2 schedules (an α/β ratio < 1 should have been used to obtain the equivalence between the shorter and conventional schedule).

Androgen deprivation (AD) has become the standard treatment in combination with RT in high risk tumors, although the duration of AD and the sequence between the two modalities has not yet been optimized (15-18). A recent randomized study (19) showed a superiority of 3-year with respect to 6-month AD in combination with 70 Gy conventional fractionation RT. However, the today standard dose delivered to the prostate is higher than 70 Gy (20) especially in high risk tumors and, therefore, the role and duration of AD should be redefined.

**02 General remarks**

This is a randomized study of hypofractionation vs. conventional fractionation RT in combination with total AD in high risk tumors.

**2.1** *Definition of high risk tumors*

The patients with high risk tumors are those with an iPSA > 20 ng/ml or GS >7, or T-stage >2c, or with the presence of at least 2 of the following clinical factors: iPSA 11-20 ng/ml, T= 2c, GS=7 (20).

**2.2** *Treatment*

The patients will be treated with a short or conventional RT course to prostate and seminal vesicles (SV), no matter of the risk of microscopic N+, in combination with 9-month total AD.

**03 Trial objectives**

**3.1** *Primary objective*

Reduction of late toxicity while maintaining the same Freedom from biochemical failure (FFBF) by hypofractionation with respect to conventional fractionation radiotherapy .

**3.2** *Secondary objective.*

Overall survival, Cause-specific survival, local, regional and distant control.

**04 Patient inclusion criteria**

**4.1** *Inclusion criteria*

Patients ≤ 80 years old, with histologically proven prostate carcinoma

WHO performance status ≤ 2

Any case with high risk tumor (as above defined)

No pelvic nodes > 1cm at the TC or RM evaluation

Previous TURP is allowed provided there is at least 6 weeks interval with the initiation of RT

Written informed consent

**4.2** *Exclusion criteria*

Previous pelvic RT or major surgery (Colo-rectal anastomosys, Total cistectomy, previous prostate surgery other than TURP, etc.)

Ulcerative proctitis

No previous malignant tumors, with the exception of the cutaneous basal cell carcinoma and other tumors cured from ≥ 5 years.

PSA>100 ng/ml.

**05 Study Design**

This is a phase III, non blinded, randomized study comparing 80 Gy conventional fractionation RT with a biologically equivalent hypofractionated course. The equivalence has been calculated by means of the linear-quadratic model using an α/β ratio of 1.5 as shown by several studies (1,5,6). The aim of this study is to determine if incidence of late toxicity is lower with the hypofractionation than with conventional fractionation, maintaining the same rate of local control/FFBF.

LHRH LHRH LHRH

0 7 67 97 102 123 187 270

Days **--------------------------------------**

Arm B___

Arm A_______

bicalutamide

**06 Therapeutic regimens**

**6.1** *Hormonal Therapy*

**6.1.1** *LHRH Analogues:* an LHRH analog depot (preferably Goserelin 10.8 g) will be administered with a subcutaneous injection at days 7, 97 and 187.

**6.1.2** *Antiandrogens:* the non-steroid antiandrogen *bicalutamide* will be orally administered at a daily dose of 50 mg for 270 days beginning at day 0 (7 days before the first LHRH analogue administration).

**6.2** *Radiotherapy*

The radiotherapy treatment will start at day 67 from the beginning of oral bicalutamide (60 days after the first LHRH analogue).

**6.2.1** *Patient data acquisition:* the treatment planning will be based on TC data acquisition. The volumes of interest (VOI) will be delineated on TC slices, taken at a minimum interval of 5 mm.

**6.2.2** *Target volumes:* The clinical target volume **(CTV)** is the prostate plus the entire SV. Planning target volume **(PTV)** = CTV + margin (according to institutional policy):

|  |  | |  | | | |
| --- | --- | --- | --- | --- | --- | --- |
|  |  |  |  |
|  |  |  |  |  |  |  |
|  |  |  |  |  |  |  |
|  |  |  |  |  |  |  |
|  |  |  |  |  |
|  |  |  |  |  |  |  |
|  |  |  |  |  |

**6.2.3** *Delineation of VOI:* The following volumes will be outlined on the planning CT: CTV, PTV, rectum from the inferior margin of the anus to the rectal- sigmoid junction, bladder, femoral heads. For rectum and bladder, where is possible the *wall* will be delineated; if the internal border of the bladder is not evident, a contour of 4 mm will be delineated.

**6.2.4** *Dose prescription:* Dose is specified to the ICRU reference point, positioned at the isocenter and/or close to the PTV center. Minimal and maximal dose to the PTV is 95% to 107% if achievable with respect to normal tissue dose-volume constraints, which have priority over the 95% dose-coverage of the PTV. Minimal dose to the CTV is 95%. Dose distribution and Dose-Volume Histograms must be accurately recorded.

**6.2.5** *Treatment technique:* Patients will receive either 3D-CRT **6.2.6** *Patient treatment position:* Supine. Personalized body-fix device with ankles support

**6.2.7** *Treatment verification:* the verification of the treatment position with respect to the DRR will be done at least twice a week by the individual institutional method (portal imaging, optic, X-ray or ultrasound device).

**07 Randomization**

All patients will receive a total AD for 9-months and will be randomly assigned to one of the following radiotherapy schedules:

**RANDOM**

Standard

80 Gy/40 F/8 wks (5 F/w)

Bicalutamide 50 mg/day

for 270 days

+

Goserelin 10,8 mg every 12 weeks for 3 times

Hypofractionated

62 Gy/20 F/5 wks (4 F/w)

**7.1** *Arm A (Control)*

**7.1.1** *PTV dose:* The PTV will receive a total dose of 80 Gy in 40 consecutive fractions of 2.0 Gy, 1 fraction per day, 5 fractions per week for 8 weeks.

**7.1.2** *Rectum dose:* maximal dose to the rectum should not be > 80 Gy. V70 and V50 (87.5% and 62.5%, respectively of 80 Gy) should not be > 30% and >50%, respectively, of the entire rectal wall.

**7.1.3** *Bladder dose:*V70 and V50 should not be > 50% and > 70%, respectively, of the entire bladder wall.

**7.1.4** *Femoral head dose:* maximal dose absorbed by the femoral heads should not be > 55 Gy (68.7% of 80 Gy).

**7.2** *Arm B (Experimental arm)*

**7.2.1** *PTV Dose:* The PTV will receive a total dose of 62 Gy in 20 fractions of 3.1 Gy, 1 fraction per day, 4 fractions per week, for 5 weeks. According to the linear-quadratic model, this dose should be at least biologically equivalent to that of the control arm, using an α/β = 1.5 Gy (4).

**7.2.2** *Rectum dose:* the maximum dose to the rectal wall should not be > 62 Gy. V54 and V39 (87% and 61%, respectively of 62 Gy) should not be > 30% and > 50% of the entire rectum wall.

**7.2.3** *Bladder dose:* V54 and V39 should not exceed 50% and 70%, respectively, of the entire bladder wall.

**7.2.4** *Femoral head dose:* the maximal dose to the femoral heads should not exceed 42 Gy (68% of 62 Gy).

**08 Evaluation**

**8.1** *Endpoints*

**8.1.1** *Freedom From Biochemical Failure (FFBF):* time interval from the randomization day to the biochemical relapse defined according to the most recent ASTRO Phoenix definition of nadir PSA +2 ng/ml (21).

**8.1.2** *Acute Toxicity:* Acute toxicity will be assessed using the RTOG/EORTC system (22) extended with a few symptoms not mentioned in the RTOG/EORTC acute radiation toxicity scale. Even a single-occasion symptom above baseline will be scored as toxicity. The AUA symptom index for BPH will be used to evaluate obstructive urinary symptoms (23). The AUA form has to be filled in by the patient (see Appendix)

**8.1.3** *Late Toxicity:* Late side effects will be assessed using a modified ("clinical") SOMA/LENT (24), in which side effects that can only be assessed by laboratory/technical investigations are eliminated. The AUA symptom index for BPH will be used to evaluate obstructive urinary symptoms. The AUA forms will be completed by the patient.

**8.1.4** *Disease-Free Survival (DFS):* time interval from the randomization day to the first clinical sign of disease progression.

**8.1.5** *Disease-Specific Survival (DSS):* time interval from the randomization day to the death for disease, with the exclusion of deaths for other diseases.

**8.1.6** *Overall Survival (OS):* time interval from the randomization day to death for any cause.

**8.2** *Timing*

**8.2.1.***Before treatment*

**a.** *Clinical Examinations*

- General examination

- WHO Performance Status (PS)

- Digital rectal examination with the evaluation of tumor diameter.

**b.** *Instrumental Examination*

- TRUS or MR for the evaluation of tumor local extension

- Pelvic CT or MR

- Multiple sextant prostate biopsy for the definition of Gleason Score

- Anorectal endoscopy for the baseline evaluation of the rectal mucosa (see

in the appendix).

- Bone scan

**c.** *Laboratory examinations*

- Baseline PSA determination

- WBC, RBC, and Platelet count, with HG determination

- Serum Testosteron and Dihydrotestosteron determination

- Histology with GS evaluation

**8.2.2** *During the radiotherapy treatment*

Acute toxicity will be assessed:

- at baseline (prior to radiation)

- once a week during radiotherapy

- 1 and 2 months after the end of radiation (where the acute period by definition terminates).

**8.2.3** *During the follow-up period*

**a.** *Instrumental, Clinical and Laboratory Examinations:*

*-* PSA determination every 3 months for 3 years and then every 6 months.

- Testosteron and Dihydrotestosteron determination every 6 months for 3 years.

- DRE every 6 months for 3 years and, then, every 12 months

- TRUS or MR every 6 months until complete tumor clearance.

- Ano-rectal endoscopy every 6 months for 2 years.

- Sextant biopsies 2 years after the end of radiotherapy.

- Evaluation of the late toxicity every 6 months for 3 years, and yearly thereafter.

**8.2.5** *Summary of the examination timing*

|  | Baseline  evaluation | During  RT | Every 3mo.  for 3 yrs, then every 6 mo. | Every 6mo.  for 3 yrs.,  then yearly | At 2 yrs. or for suspected  Progression |
| --- | --- | --- | --- | --- | --- |
| **Clinical** | X |  |  | X |  |
| General | X |  |  | X |  |
| PS | X |  |  | X |  |
| DRE | X | X (RT end) |  | X |  |
| Acute Tox |  | X (weekly) |  |  |  |
| Late Tox |  |  |  | X |  |
|  |  |  |  |  |  |
| **Instrumental** |  |  |  |  |  |
| TRUS/RM | X |  |  | X (till CR) | X |
| Sextant biopsy | X |  |  |  | X |
| Ano-rectal endo | X |  |  | X (for 2 yrs) | X |
| Pelvic CT/MR | X |  |  |  | X |
| Bone scan | X |  |  |  | X |
|  |  |  |  |  |  |
| **Laboratory** |  |  |  |  |  |
| PSA | X | X (baseline)  X (RT end) | X |  | X |
| Histology (GS) | X |  |  |  | X |
| Blood count | X | X (weekly) |  |  |  |
| Testosteron/  dihidrotestosteron | X |  |  | X (for 3 Yrs) |  |

**09 Disease monitoring and treatment of progressive disease**

**9.1** *Disease monitoring*  In case of a PSA rising and/or suspected clinical local (new or progressive palpable prostate nodule) or distant failure (bone pain, low extremity edema, unjustified dyspnea, etc.):

- Bone scan

- CT of the thorax, abdomen and pelvis

- Bone CT or MR to confirm the bone scan data

- TRUS or MR of the prostate

- Prostate biopsy

- Choline PET scan (optional).

**9.2** *Treatment of progressive disease*

The timing and nature of treatment in case of progressive disease will be left at the decision of the individual physician.

**10 Statistical considerations, sample size, data analysis and reporting**

The main objective of the trial is to investigate the late toxicity and freedom from biochemical failure.

This is a single-institution, prospective, phase III randomized trial designed to randomize patients with high risk prostate cancer to receive 80 Gy in 40 fractions in 8 weeks at 2.0 Gy per fraction (arm A, conventional fractionation) or 62 Gy in 20 fractions in 5 weeks (4 fractions per week) at 3.1 Gy per fraction (arm B, hypofractionation) to the prostate. The two arms were hypothesized to be isoeffective with regard to tumor control. However, with regard to late complications, the hypofractionation regime of this trial should be equivalent to 75 Gy given at 2.0 Gy per fraction that is lower than 80 Gy given in the conventional fractionation arm. From the data available in the literature and using the Lyman-Burman-Kutcher (LBK) model the 3-year rates of grade 2 or higher late rectal damage were estimated to be approximately 29% and 12% after 80 or 75 Gy, respectively, delivered at 2.0 Gy per fraction. On the basis of an 80% power to detect a significant difference (p<0.05, two sided), 84 patients were required in each arm (168 patients in total).

Late toxicity and outcome will be analyzed after median follow-up has reached 3 years.

**11 Ethical – legal considerations**

The patients will be entered in this trial only after protocol approval by the ethics committee. Written informed consent is required for participation. The legal coverage of this trial is a the responsibility of Regina Elena Cancer Institute.

**12 Administrative issues**

**12.1** *Planning documentation*

For future analyses, the following planning data should be recorded:

- Dose-volume histograms of all delineated structures.

- Absolute volumes of delineated structures.

# 13. References

01. Brenner DJ, Hall EJ. Fractionation and protraction for radiotherapy of prostate carcinoma. Int J Radiat Oncol Biol Phys 1999; 43: 1095-1101.

02. King CR, Mayo CS. Is the prostate α/β ratio of 1.5 from Brenner & Hall a modeling artifact? Int J Radiat Oncol Biol Phys 2000; 47: 536-537.

03. Brenner DJ, Hall EJ. In response to drs. King and Mayo: low α/β values for prostate appear to be independent of modeling details. Int J Radiat Oncol Biol Phys 2000; 47: 538-539.

04. Fowler J, Chappell R, Ritter M, et al. Is alpha/beta for prostate tumours really low? Int J Radiat Oncol Biol Phys 2001; 50: 1021-1031.

05. King CR, Fowler JF. A simple analytic derivation suggests that prostate cancer α/β ratio is low. Int J Radiat Oncol Biol Phys 2001; 51: 213-214.

06. Brenner DJ, Martinez AA, Edmundson GK, et al. Direct evidence that prostate tumours show high sensitivity to fractionation (low alpha/beta ratio), similar to late responding normal tissue. Int J Radiat. Oncol. Biol. Phys. 2002; 52: 6-13.

07. Wang JZ, Guerrero M, Li XA. How low is the α/β ratio for prostate cancer? Int J Radiat. Oncol. Biol. Phys. 2003; 55: 194-203.

08. Kal HB, Van Gellekom MPR. How low is the α/β ratio for prostate cancer? Int J Radiat Oncol Biol Phys 2003; 57: 1116-1121.

09. Lloyd-Davies RW, Collins CD, Swan AV. Carcinoma of the prostate treated by radical external beam radiotherapy using hypofractionation. Twenty-two years’ experience (1962-1984). Urology 1990; 36: 107-111.

10. Logue JP, Hendry JH. Hypofractionation for prostate cancer. Int J Radiat Oncol Biol Phys 2001; 49: 1522.

11. Kupelian PA, Reddy CA, Carlson TP et al. Preliminary observations on biochemical relapse-free survival rates after short-course intensity-modulated radiotherapy (70 Gy at 2.5 Gy/fraction) for localized prostate cancer. Int J Radiat Oncol Biol Phys 2002; 53: 904-912.

12. Livsey JE, Cowan RA, Wylie JP et al. Hypofractionated conformal radiotherapy in carcinoma of the prostate: five-year outcome analysis. Int J Radiat Oncol Biol Phys 2003; 57: 1254-1259.

13. Lukka H, Hayter C, Julian JA, et al. Randomized trial comparing two fractionation schedules for patients with localized prostate cancer. J Clin Oncol 2005;23:6132-6138.

14. Yeoh EE, Holloway RH, Fraser RG, et al. Hypofractionated versus conventionally fractionated radiation therapy for prostate cancer: Updated results of a phase III randomized trial. In J Radiat Oncol Biol Phys 2006;66: 1072-1083.

15. Bolla M, Collette L, Blank L, et al. Long-term results with immediate androgen suppression and external irradiation in patients with locally advanced prostate cancer (an EORTC study): a phase III randomized trial. Lancet 2002;360:103-108.

16. Pilepich MV, Winter K, John MJ et al. Phase III radiation therapy oncology group (RTOG) trial 86-10 of androgen deprivation adjuvant to definitive radiotherapy in locally advanced carcinoma of the prostate. Int J Radiat Oncol Biol Phys 2001; 50: 1243-1252.

17. Pilepich MV, Winter K, Lawton CA et al. Androgen suppression adjuvant to definitive radiotherapy in prostate carcinoma. Long term results of phase III RTOG 8531. Int J Radiat Oncol Biol Phys 2005;61: 1285-1290

18. Hanks GE, Pajak TF, Porter A, et al. Phase III trial of long-term adjuvant androgen deprivation after neoadjuvant hormonal cytoreduction and radiotherapy in locally advanced carcinoma of the prostate: The Radiation Therapy Oncology Group Protocol 92-02. J Clin Oncol 2003;21: 3972-3978.

19. Bolla M, van Tienhoven G, de Reijke TM et al. **Concomitant and adjuvant androgen deprivation (ADT) with external beam irradiation (RT) for locally advanced prostate cancer: 6 months versus 3 years ADT—Results of the randomized EORTC Phase III trial 22961. J Clin Oncol**  2007; 25(Suppl 18): 5015 (abstr.).

20. Prostate cancer. In: National Comprehensive Cancer Network Clinical Practice Guidelines in Oncology, version 2, 2005. Jenkinstown, PA: National Comprehensive Cancer Network, June 2006.

21. Roach III m, Hanks G, Thames H Jr, et al. Defining biochemical failure following radiotherapy with or without hormonal therapy in men with clinically localized prostate cancer: recommendations of the RTOG-ASTRO Phoenix Consensus Conference. Int J Radiat Oncol Biol Phys 2006; 65: 965-974.

22. Cox JD, Stetz J, Pajak TF. Toxicity criteria of the Radiation therapy Oncology group (RTOG) and the European organization for research and treatment of cancer (EORTC). Int J Radiat Oncol Biol Phys 1995; 31: 1341-1346.

23. Barry MJ, Fowler FJ Jr, O’Leary MP et al. The American Urological Association symptom index for benign prostatic hyperplasia. The measurement Committee of the American Urological Association. J Urol 1992; 148: 1549-1557.

24. Pavy JJ, Denekamp J, Letschert J et al. Late effects toxicity scoring: the SOMA scale. Int J Radiat Oncol Biol Phys 1995; 31: 1043-1049.

25. Zelefsky MJ, Leibel SA, Gaudin PB, et al. Dose escalation with three-dimensional conformal radiation therapy affects the outcome in prostate cancer. Int J Radiat Oncol Biol Phys 1998; 41: 491-500.

26. Storey MR, Pollack A, Zagars G et al. Complications from radiotherapy dose in prostate cancer: preliminary results of a randomised trial. Int J Radiat Oncol Biol Phys 2000; 48: 635-642.

27. Lemeshow S, Hosmer DW Jr, Klar J, Lwanga SK. Adequacy of sample size in health studies. 1^st^ ed. Chichester (UK): John Wiley & Sons ltd.; 1990.
